# Oncocytic Lesions of Salivary Glands: Morphological, Immunohistochemical, and Molecular Findings

**DOI:** 10.7759/cureus.59328

**Published:** 2024-04-29

**Authors:** Riddhi Parmar, Amankumar N Kalaria, Keval A Patel

**Affiliations:** 1 Department of Pathology, All India Institute of Medical Sciences, Rajkot, Rajkot, IND; 2 Department of Pathology, Swaminarayan Institute of Medical Sciences & Research, Kalol, IND; 3 Department of Pathology, Gujarat Medical Education & Research Society (GMERS) Medical College, Vadnagar, IND

**Keywords:** molecular, immunohistochemistry, oncocytic lesions, neoplasm, salivary gland

## Abstract

The fifth edition of the World Health Organization (WHO) classification introduces new diagnostic methods based on genetic alterations, providing insight into the molecular basis of lesions. As a result, the classification system has evolved, new entities have been introduced, and existing entities have been reclassified. Oncocytic lesions of salivary glands are a group of neoplastic conditions characterized by the presence of oncocytic cells. These lesions present a diagnostic challenge due to their overlapping histological features. Therefore, a comprehensive evaluation, including morphological, immunohistochemical, and molecular analysis, is crucial for accurate diagnosis and appropriate management. Accurate classification of salivary gland pathologies is essential for selecting the appropriate treatment methods and predicting outcomes. The introduction of new therapeutic approaches, such as targeted therapies for malignant salivary gland tumors, has improved patient outcomes. However, to effectively implement these therapies in clinical practice, a clear classification of lesions is necessary.

## Introduction and background

Among head and neck tumors, salivary gland tumors (SGTs) account for only 5% of cases [1-4]. The fifth edition of the World Health Organization (WHO) Classification of Head and Neck Tumors identifies 35 different salivary gland pathologies [2]. These tumors can exhibit benign, low-grade, or high-grade malignant behavior. Morphological similarities between tumor types and variations within a single tumor can make the evaluation challenging. Hybrid lesions, dedifferentiation, and malignant transformation further complicate the morphological assessment. While hematoxylin and eosin (H&E)-stained sections are typically used for diagnosis, immunohistochemical markers play an important role in defining cellular differentiation. The major salivary glands in the upper respiratory system are the parotid, submandibular, and sublingual glands. They consist of serous, mucous, and mixed acini, as well as intercalated, striated duct, and excretory ducts. Myoepithelial cells contract to push secretions out of acini and intercalated channels, while basal cells support the structure and function of striated and excretory ducts. Salivary gland neoplasms start in luminal cells, which are made up of acinar/ductal epithelial cells, and/or abluminal cells, which are made up of myoepithelial/basal cell lineage. The abluminal cells in the salivary gland are located on the outer surface of the glandular epithelium, providing support, protection, and regulating substance transport. Myoepithelioma, acinic cell carcinoma (AciCCA), and salivary gland duct carcinoma are monophasic tumors that arise from only one cellular component, while pleomorphic adenoma, epithelial-myoepithelial carcinoma, and adenoid cystic carcinoma are biphasic tumors that originate from both luminal and abluminal cells.

Many reactive and neoplastic salivary gland lesions exhibit oncocytic cells with large, granular eosinophilic cytoplasm rich in mitochondria. Oncocytic lesions of the salivary gland are classified as nodular oncocytic hyperplasia, oncocytoma, and oncocytic carcinoma in the fifth edition of the WHO Classification of Head and Neck Tumors. Nodular oncocytic hyperplasia is a non-neoplastic epithelial lesion typically found in the parotid gland in individuals aged 50-60 years.

Oncocytoma, a rare benign tumor composed of oncocytes, which are large, eosinophilic cells with abundant mitochondria arranged in a solid, tubular, and trabecular pattern, may show clear cell changes. It is typically involved in the superficial lobe of the parotid gland and is well-circumscribed. It usually affects the superficial lobe of the parotid gland and is well-defined. It is characterized by the proliferation of monotonous oncocytic cells and is encapsulated. Malignancy of oncocytes is indicated by the absence of a capsule, perineural-vascular-soft tissue invasion, atypia, and increased mitotic activity [4]. Oncocytic carcinoma is a locally aggressive tumor that invades surrounding tissue and shows regional lymphatic involvement, making it diagnostic. Perineural invasion may also be observed [5-7]. Warthin tumor, oncocytic cystadenoma, mucoepidermoid carcinoma (MEC), acinic cell carcinoma (AciCCA), mammary analog secretory carcinoma (MASC), and metastatic renal cell carcinoma (RCC) are other monomorphic oncocytic neoplasms that present diagnostic challenges. Salivary gland duct carcinoma (SDCA), high-grade MEC, metastatic squamous cell carcinoma (SCC), metastatic adenocarcinoma, and metastatic melanoma are examples of pleomorphic oncocytic neoplasms [8]. It is important to consider cutaneous SCC and melanoma metastases, as well as intraparotid lymph node spread with retrograde flow in hypopharyngeal and laryngeal carcinomas, as potential differential diagnoses [9]. The use of the latest classification and literature findings can serve as a helpful tool in developing a diagnostic algorithm for this group of lesions. Oncocytomas are generally considered to be benign and have a favorable prognosis; oncocytic carcinomas, oncocytic mucoepidermoid carcinomas, and other oncocytic variants of carcinoma are more aggressive and have a higher risk of recurrence and metastasis. Therefore, accurate diagnosis and appropriate management of these tumors are crucial for optimal patient care. This article elaborates on the details of histopathology, immunohistochemistry (IHC), and molecular findings that aid in the diagnosis of oncocytic lesions of the salivary gland.

## Review

Warthin’s tumor

**Figure 1:**
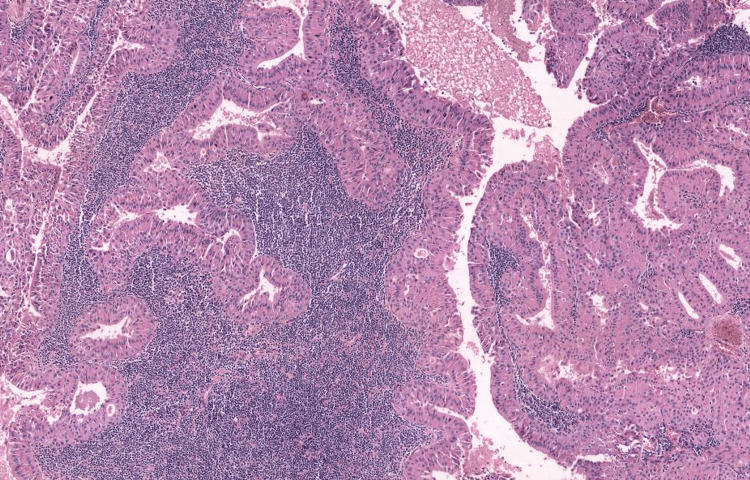
Show a well-circumscribed tumor with a papillary architecture. The papillae in the case of Warthin's tumor are lined by bilayer oncocytic epithelial cells and surrounded by a lymphoid stroma (H&E, x200). Note: Author’s own patient image. H&E: hematoxylin and eosin.

Warthin's tumor, also known as papillary lymphomatous cystadenoma or adenolymphoma, is the second most common benign tumor of the parotid gland, accounting for about 15% of cases [10,11]. It primarily affects male patients in their sixth and seventh decades of life. Unlike other benign tumors of the salivary glands, Warthin's tumor often shows bilateral involvement of the parotid gland, with approximately 90% of cases occurring in the superficial lobe. Grossly, Warthin’s tumors are well-circumscribed, spherical to oval masses. The cut surface shows solid and multiple cysts with papillary projection filled with mucoid or brown fluid. Histologically, the tumor consists of oncocytic epithelial cells arranged in rows (papillary cystic structure) surrounded by cystic spaces and a lymphoid stroma with germinal centers [12] (Figure 1).

Treatment typically involves surgery to remove part or all the parotid gland while preserving the facial nerve [13]. Malignant transformation is rare, occurring in only 0.1% of cases, and usually involves the epithelial component of the tumor. Postoperative recurrence is extremely rare. MAML2 fusion is absent in Warthin’s tumors, present in mucoepidermoid carcinoma.

Oncocytoma

**Figure 2:**
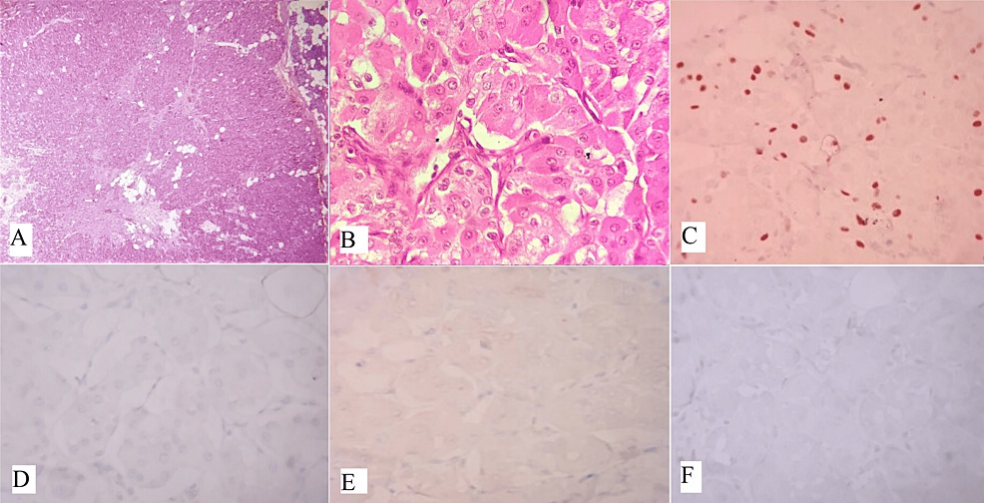
(A) Oncocytoma is composed of a sheet and solid cluster of oncocytic cells (H&E, x100), (B) shows oncocytic cells with uniform, predominantly centrally located nuclei and abundant eosinophilic cytoplasm (H&E, x400), IHC shows tumor cells strongly positive for (C) P63 (x400) and negative for (D) DOG1 (x400), (E) AR (x400), and (F) S100 (x400). Note: Author’s own patient images. IHC: immunohistochemistry; H&E: hematoxylin and eosin.

Oncocytomas are uncommon benign tumors that account for less than 1% of all salivary gland neoplasms [14]. They are characterized by oncocytes, which are cuboidal to columnar epithelial cells with an abundant eosinophilic cytoplasm resulting from an excess of mitochondria. Typically, oncocytomas are asymptomatic, well-defined, solitary masses that are painless and usually measure 3-4 cm in diameter, although they can reach up to 7 cm. In rare cases, they may cause pain or discomfort and can also occur as multiple or bilateral tumors [14]. Oncocytomas typically appear as solid, well-defined, tan to red-brown nodular masses. They are distinguished from oncocytic hyperplasia by fibrous encapsulation. Microscopically, oncocytomas show densely packed oncocytes in solid clusters or cords with a fibrovascular stroma (Figure 2A). The cells have large, cuboidal to columnar shapes with prominent eosinophilic cytoplasm and round nuclei (Figure 2B). Scattered lumina of varying sizes may contain eosinophilic secretions. The eosinophilia can vary, resulting in a mixture of light and dark-stained cells. Occasionally, oncocytomas exhibit extensive cystic changes. Cytokeratin stains positively in oncocytomas, and basal cells can be identified with p63 staining. However, these basal cells are not visible under routine light microscopy. Immunohistochemistry (IHC) shows the tumor cells are positive for P63 (Figure 2C), negative for DOG1 (Figure 2D), AR (Figure 2E), and S100 (Figure 2F). Table 1 displays the IHC markers utilized for distinguishing oncocytic variants of salivary gland tumors [8]. Oncocytomas do not show positive staining for specific myoepithelial markers like smooth muscle actin, calponin, S-100 protein, and glial fibrillary acidic protein (GFAP) [15].

**Table 1:** IHC markers used to differentiate oncocytic variants of salivary gland tumor. +: positive, −: negative, −/+: negative/positive, IHC: immunohistochemistry. Note: Adapted from Reference [8].

	P63	P40	S100	Mammaglobin	Sox10	DOG1	GATA3	AR
Warthin & oncocytoma	+	−	−	−	−	−	−	−
Acinic cell carcinoma	−	−	−	−	+	+	−	−
Secretory carcinoma	−	−	+	+	+	−	+	−
Mucoepidermoid carcinoma	+	+	−	−	−/+	−/+	−	−
Salivary duct carcinoma	−	−	−	−/+	−	−	+	+
Granular cell tumor	−	−	+	−	+	−	−	−

Oncocytic carcinoma

**Figure 3:**
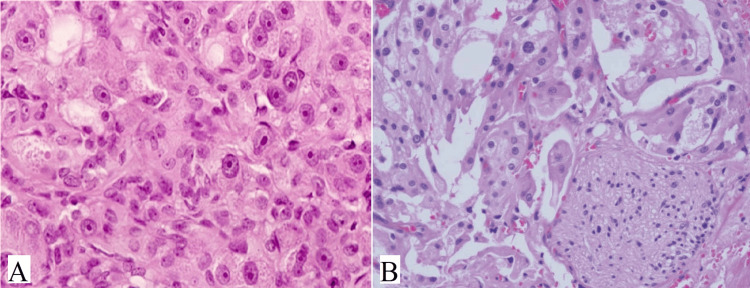
(A) Shows tumor cells of oncocytic carcinoma having nuclear pleomorphism, a high nuclear-to-cytoplasmic ratio, prominent nucleoli, and eosinophilic granular cytoplasm (H&E, x400). (B) The tumor cells invade surrounding tissues with perineural invasion seen (H&E, x400). Note: Author’s own patient image. H&E: hematoxylin and eosin.

Oncocytic carcinoma is a rare tumor that originates in salivary glands. It is characterized by the proliferation of malignant oncocytes with adenocarcinomatous architectural phenotypes. This tumor can arise de novo or be associated with pre-existing oncocytoma [16]. Patients typically present with painless, slow-growing swellings. The tumor grossly appeared as a grayish-yellow, irregular well-defined mass. Oncocytic carcinoma is characterized by large polyhedral cells with abundant granular eosinophilic cytoplasm and round to oval vesicular nuclei, sometimes with double nuclei and prominent nucleoli (Figure 3A). These cells formed organoid nests and trabeculae with infiltrative features. Tubular structures, nuclear pleomorphism, vascular, and perineural invasion (Figure 3B) may also be observed.

To identify the malignant nature of oncocytic carcinoma, certain criteria should be considered. These include lack of encapsulation, frequent mitoses and cellular pleomorphism, perineural, intravascular, or lymphatic invasion, and regional or distant metastases [17]. Local recurrence is also a characteristic of oncocytic carcinoma. According to WHO Histological Typing of Salivary Gland Tumors (2017), the diagnosis of oncocytic carcinoma requires the identification of oncocytes and the presence of cellular and nuclear pleomorphism, local infiltration, and metastasis. Clinically, the presence of recurrences and lymphatic involvement suggests a malignancy. Approximately 20% of oncocytic carcinomas exhibit necrosis [18]. In certain instances, benign oncocytomas may lack a well-defined capsule and exhibit a diffuse infiltrative appearance, possibly due to widespread oncocytic changes or the involvement of multiple small nodules [19]. To differentiate between benign and malignant oncocytic tumors, it is crucial to note the absence of malignant histological characteristics like perineural invasion, vascular invasion, and lymphatic spread.

Oncocytic variants of mucoepidermoid carcinoma

**Figure 4:**
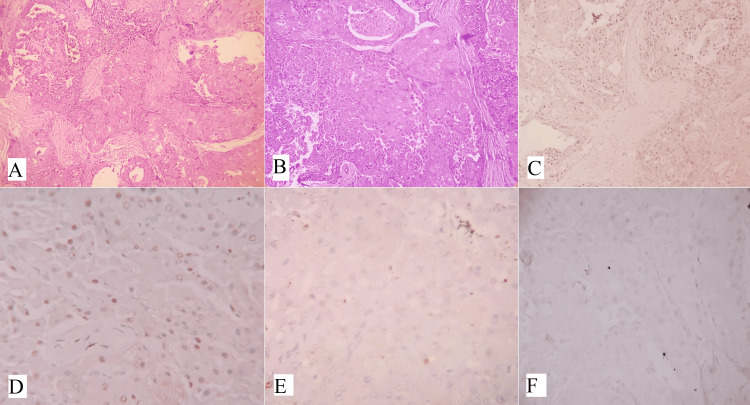
(A) Shows a cystic structure lined by mucous cells and solid nests of epidermoid cells characterized by mucoepidermoid carcinoma (H&E, x100). (B) Shows extensive oncocytic differentiation (H&E, x200). (C) and (D) IHC shows tumor cells are positive for (C) GATA3 (x400) and (D) P63 (x400). (E) S100 (x400) and (F) AR (x400) are negative. Note: Author’s own patient images. IHC: immunohistochemistry; H&E: hematoxylin and eosin.

Oncocytic variants of mucoepidermoid carcinoma (OMEC) are rare and characterized by the presence of more than 50% oncocytic cells. However, oncocytic metaplasia in mucoepidermoid carcinoma is common [20,21]. Oncocytic change occurs due to alterations in the mitochondria and is characterized by enlarged cells with abundant eosinophilic granular cytoplasm [22]. Oncocytic neoplasms account for 1% of parotid neoplasms, with only 11% being malignant [23]. Diagnosing OMEC can be challenging if typical mucoepidermoid carcinoma cells are limited to small foci or absent. OMEC tumors display oncocytic cells arranged in nests, trabeculae, and sheets, interspersed with desmoplastic stroma and chronic inflammatory cells. Cytologically, the oncocytic cells are round to polygonal, with centrally placed nuclei, prominent nucleoli, and abundant granular eosinophilic cytoplasm. The majority of the tumors were surrounded by a fibro-collagenous capsule (Figures 4A, 4B). Immunohistochemical staining shows positivity for CK7, GATA3 (Figure 4C), P63 (Figure 4D) and negativity for S100 (Figure 4E), AR (Figure 4F), CD10, and renal cell carcinoma markers. Mucin can be detected using a mucicarmine stain. Recent studies have shown that p63 exhibits nuclear staining in OMEC, with a peripheral pattern in oncocytoma and oncocytic carcinomas. Fluorescent in situ hybridization (FISH) testing can detect MECT1-MAML2 fusions, as MEC frequently involves a t(11,19) (q21;p23) translocation [20,24].

Management of salivary gland tumors depends on several factors. Partial superficial parotidectomy is recommended for benign tumors and low-grade MEC [25]. Postoperative radiation may be considered based on specific risk factors such as close or positive margins, perineural invasion, lymph node involvement, or high-grade tumors, and advanced stages T3 and T4. Chemotherapy is generally not part of the standard treatment for MEC [25]. Patients should be regularly followed up, with a decreasing frequency of follow-up visits over time. Overall, MEC has a good prognosis, and the OMEC variant does not affect the prognosis [24].

Acinic cell carcinoma

**Figure 5:**
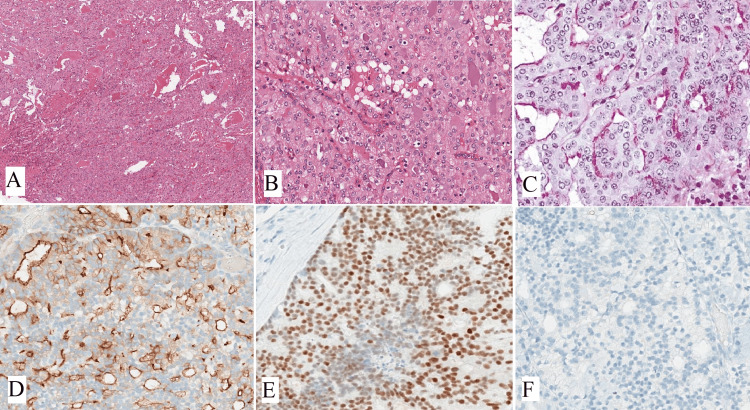
(A) Shows a tumor composed of sheets of typical oncocytic cells with bland nuclei and abundant eosinophilic cytoplasm (H&E, x100). (B) Focal areas of abundant eosinophilic cytoplasm (H&E, x400). (C) Shows fine PAS-positive cytoplasmic granules toward the luminal side of the tumor cells, case of oncocytic variant ACC (PAS-D, x400). (D) IHC shows tumor cells are membranous positive for DOG1 (x400), (E) nuclear staining positivity for SOX-10 (x400), and negative for (F) mammaglobin (x400). Note: Author’s own patient images. PAS-D: periodic acid-Schiff, IHC: immunohistochemistry, H&E: hematoxylin and eosin.

Acinic cell carcinoma is a rare type of tumor that occurs in the parotid gland, making up about 2.5-7% of all parotid gland tumors. The tumor is composed of various cell types, including acinar cells, vacuolated cells, intercalated cells, non-specific glandular cells, and clear cells. These tumors can exhibit various architectural patterns, including solid, solid-lobular, acinar-microcystic, papillary cystic, tubuloductal, follicular, or macrocystic, and may show dedifferentiation. Neoplastic acinar cells resemble the polyhedral cells found in normal acini and have abundant finely granular cytoplasm, which can appear amphophilic, pale eosinophilic, or basophilic, with a nucleus that is eccentrically located (Figures 5A, 5B). Diastase-treated periodic acid-Schiff (PAS-D) stain demonstrated numerous fine PAS-positive cytoplasmic granules toward the luminal side of the tumor cells, supporting the diagnosis of the oncocytic variant of acinic cell carcinoma (Figure 5C). Solid and pseudo glandular growth patterns, along with clear cells, can be observed in both acinic cell carcinomas and oncocytomas. Although clear cells are an infrequent finding in acinic cell carcinoma, their presence may cause a histological overlap with the clear cell variants of oncocytoma [26]. The clear cells in acinic cell carcinoma are negative for glycogen and PTAH in contrast to oncocytoma, where the clear cells are glycogen and focally PTAH positive. Furthermore, it has been noted that acinic cell carcinomas lack basal or myoepithelial cells. Recent research utilizing p63 as a marker has revealed the presence of basal cells in oncocytic lesions of the salivary gland [27-29]. Weiler et al. [26] suggest that the presence of a diffuse distribution of basal cell component, as indicated by positive staining with p63 and CK5/6 antibodies, supports a diagnosis of oncocytoma, as opposed to acinic cell carcinoma, which does not contain basal cells. This basal cell component is not readily identifiable in standard H&E sections, necessitating the use of immunostains to differentiate between these two types of lesions. Immunohistochemical markers, such as DOG1 (Figure 5D) and SOX10 (Figure 5E), were positive in acinar and intercalated duct cells. Acinic cell carcinoma is usually negative for mammaglobin (Figure 5F), which can help distinguish it from secretory carcinoma. The genetic profile of acinic cell carcinoma has been studied, and alterations in the PI3K pathway have been reported. However, the significance of these findings in terms of the biology and treatment of tumors remains unknown.

Salivary duct carcinoma

**Figure 6:**
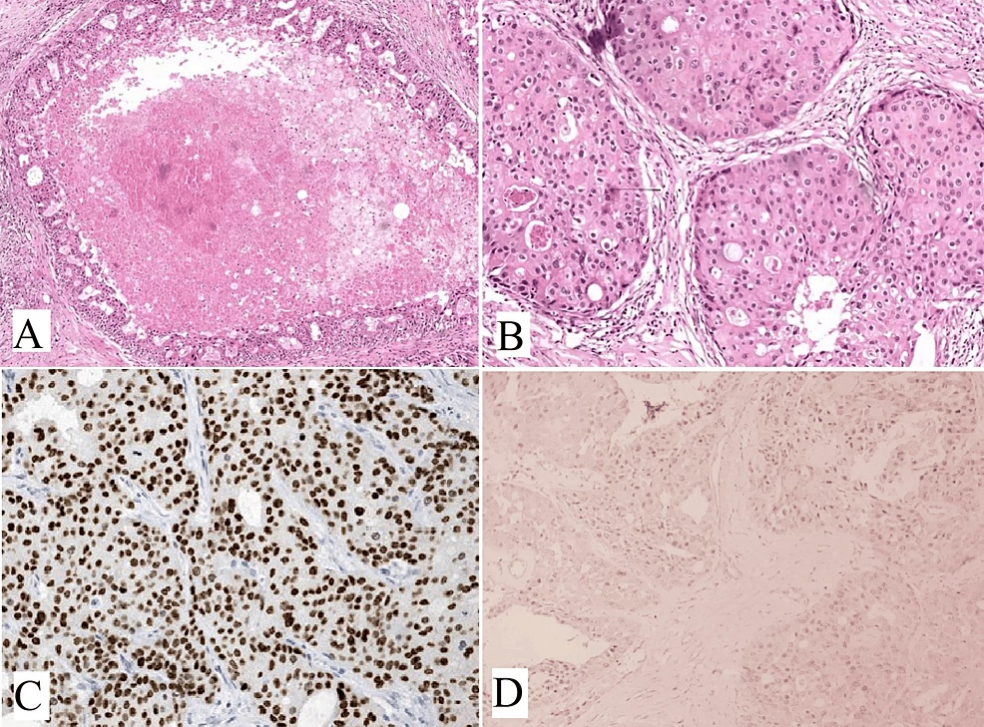
SDC shows tumor cell proliferation with central necrosis resembling breast ductal carcinoma, displaying a comedo-type pattern. (A) and (B) The tumor cells exhibit large pleomorphic nuclei with prominent nucleoli and abundant eosinophilic cytoplasm (H&E, x400). (C) Diffuse and strong positive immunohistochemical staining for androgen receptor (x400) and (D) GATA 3 (x200). Note: Author’s own patient images. SDC: salivary duct carcinoma; H&E: hematoxylin and eosin.

SDC is an aggressive type of cancer that resembles high-grade mammary ductal carcinoma. SDC is characterized by tumor cells with comedo necrosis and a cribriform pattern (Figure 6A). Tumor cells exhibit large pleomorphic nuclei with prominent nucleoli and abundant eosinophilic cytoplasm [30]. SDC typically has more cellular atypia and mitoses compared to breast carcinoma [31]. There are different variants of SDC, but they usually appear in combination with the typical features of SDC [32-34]. An oncocytic variant of SDC having >50% components shows oncocytic changes characterized by enlarged cells with abundant eosinophilic granular cytoplasm and a centrally placed nucleus (Figure 6B). Sarcomatoid, invasive micropapillary, and rhabdoid SDC have a poorer prognosis than conventional SDC [33-35]. IHC shows a strong and diffuse expression of the androgen receptor (AR) is a characteristic feature of SDC (Figure 6C). Positive staining for GATA3 (Figure 6D) or GCDFP can also aid in the diagnosis [35,36]. Recent studies have shown that over 90% of SDC cases express AR. It is important to carefully exclude other entities, such as high-grade mucoepidermoid carcinoma when diagnosing "AR-negative SDC" [36]. The expression of p63 can help differentiate mucoepidermoid carcinoma from SDC. Approximately 70% of SDC cases in both men and women show diffuse nuclear staining for AR. Estrogen receptors and progesterone receptors are negative. About 25-30% of cases have high expression of ERBB2 (HER2).

Genetic analysis has identified AR copy-number gain and splice variants, as well as ERBB2 gene amplification in SDC. Based on the biomarker profile of SDC, targeted therapies focusing on AR and HER2 have been introduced. Small cohort studies have shown promising results and larger studies from Japan and the Netherlands have provided further evidence.

Immune checkpoint inhibitor therapy has shown limited potential for therapeutic benefit in SDC [37]. The majority of salivary duct carcinoma cases are microsatellite stable and have a low tumor mutational burden. There is inconsistent data on PD-L1 expression. Research on the use of the immune checkpoint inhibitor pembrolizumab for advanced salivary gland carcinoma has shown a 12% response rate, with one case of salivary duct carcinoma included in the study group [38,39]. Recent molecular analyses have identified additional genetic alterations that can be targeted with drugs, including PIK3CA, HRAS, BRAF, PTEN, NTRK, and RET.

Secretory carcinoma

Secretory carcinoma is a rare type of cancer that may be under-reported or under-recognized owing to its recent recognition. There have been 39 reported cases of secretory carcinoma of the salivary gland, with six cases from India and 33 cases from Western countries [40]. It commonly occurs in adults, with a male-to-female ratio of 1.5:1 and a wide age range from seven to 88 years; a mean of 46.5 years. The majority of cases develop in the parotid gland, followed by the submandibular gland, minor salivary glands, and the airway system. Secretory carcinoma typically presents as a painless, slow-growing mass.

**Figure 7:**
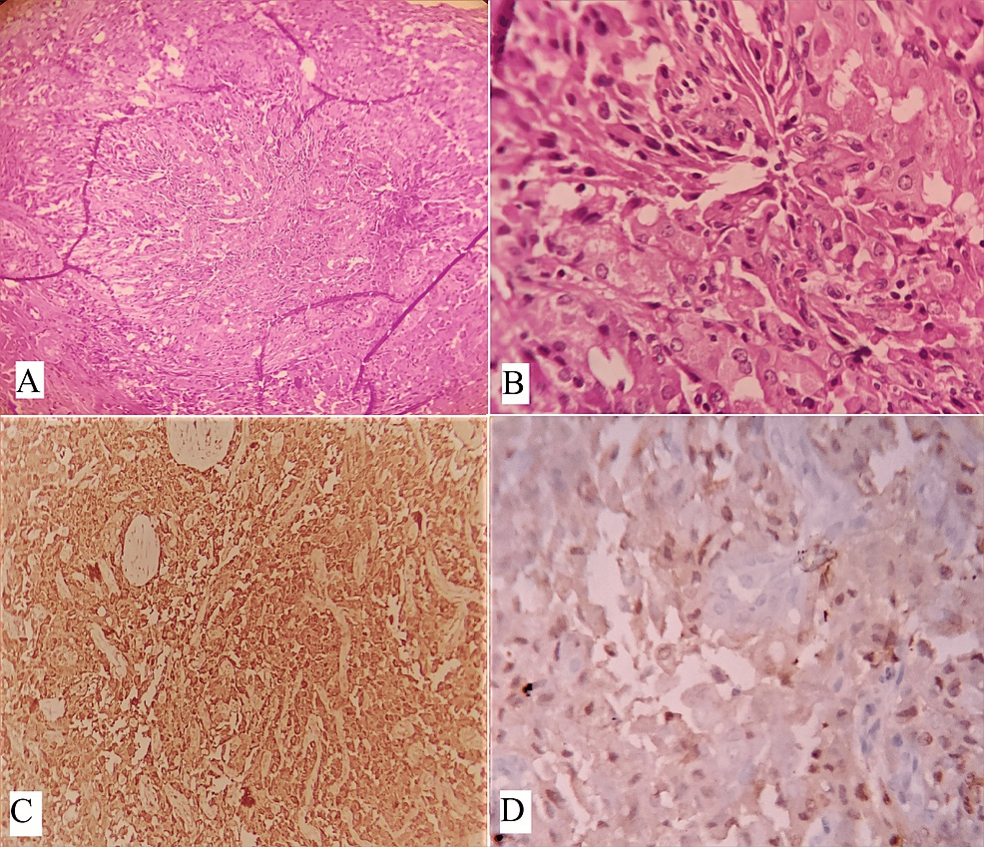
(A) Shows tumors arranged as cribriform and cluster patterns (H&E, x100), (B) shows neoplastic cells having mildly pleomorphic round to oval nuclei with fine chromatin and few with small distinctive nucleoli having abundant pale eosinophilic, granular to vacuolated cytoplasm in a case of secretory carcinoma (H&E, x400), IHC shows tumor cells strong and diffusely positive for (C) mammaglobin (x200) and (D) S100 (x400). Note: Author’s own patient images. IHC: immunohistochemistry; H&E: hematoxylin and eosin.

Grossly, secretory carcinoma appears as a solitary, well-circumscribed mass with a white-gray, brown, or yellow-cut surface. Cytomorphological features of secretory carcinoma may resemble those of other tumors, such as acinic cell carcinoma and benign neoplasms of the salivary glands. Diagnosis can be challenging without ancillary studies, and a definitive diagnosis can only be made through histological examination and IHC studies of the excised tumor. The cellular architecture of secretory carcinoma includes various arrangements, such as tight and loosely cohesive clusters, sheets, papillary groups, single cells, naked nuclei, and transgressing vessels. Neoplastic cells are round to polygonal in shape, with moderate-to-abundant cytoplasm containing vacuoles and fine cytoplasmic granules [41]. The nuclei are round to oval, with minimal irregularity and one to two prominent nucleoli (Figures 7A, 7B).

It shares similar growth patterns with acinic cell carcinoma but can be distinguished by its multivacuolated eosinophilic cytoplasm, presence of mucin, and absence of true zymogen granules. Papillary cystic architecture, which is rare in acinic cell carcinoma, is more common in secretory carcinoma. Immunohistochemistry can also aid in the diagnosis, as secretory carcinoma is mammaglobin (Figure 7C) and S100 (Figure 7D) positive but negative for DOG1, while acinic cell carcinoma shows the opposite staining pattern [42]. High-grade transformation, characterized by nuclear atypia, necrosis, and a lack of secretions, has been reported in some cases. It is characterized by a specific chromosomal translocation resulting in the ETV6-NTRK3 fusion product, which can be detected using a FISH break-apart probe for ETV6 [43,44].

Acinic cell carcinoma is the most common differential diagnosis; however, other possibilities include polymorphous low-grade adenocarcinoma, papillary cystadenocarcinoma, mucoepidermoid carcinoma, and low-grade intraductal carcinoma. Cases with high-grade transformation can mimic salivary duct carcinoma (SDC), but the expression of androgen receptor or HER-2/neu and negative staining of S100 protein can help differentiate between the two [45]. A definitive diagnosis can be made based on morphological patterns and immunohistochemistry.

Metastatic renal cell carcinoma

Metastatic renal cell carcinoma in the salivary glands is challenging to distinguish from clear cell oncocytoma based solely on histopathological features. Both tumors had a similar architectural pattern and clear cell component. Clear cell renal cell carcinoma contains accumulated droplets of glycogen, phospholipids, and neutral lipids, resulting in a clear cytoplasm. Glycogen can be demonstrated by a periodic acid-Schiff (PAS) stain, while neutral lipids can be identified with an oil-red O stain. Clear cell RCC may also contain cells with granular eosinophilic cytoplasm. Infiltrative growth, cellular and nuclear pleomorphism, and prominent stromal vascularity favor the diagnosis of clear cell renal cell carcinoma [15]. Blood lakes, which are pseudolumina-filled with fresh red blood cells, were exclusively observed in cases of metastatic RCC [29]. Salivary gland oncocytic tumors and metastatic RCC can be effectively differentiated using p63 immunohistochemical staining [46]. Oncocytic carcinomas and benign oncocytic tumors show widespread p63 nuclear positivity in their basal cells, predominantly located at the periphery of the tumor cell nests, while metastatic RCC does not. CD10 and renal cell carcinoma antigens can also be used for immunoreactivity in clear cell renal cell carcinoma [29]. Clinical and imaging studies should be done to rule out a primary renal tumor if there is uncertainty.

While standard diagnostic approaches have proven to be effective, a better understanding of the cellular and molecular mechanisms of salivary gland neoplasms is essential. The three novel diagnostic tools that have revolutionized the characterization of SGN are immunohistochemistry (IHC) and fluorescent in situ hybridization (FISH), which detect specific DNA sequences in tissue samples. It enables the visualization of genetic alterations, such as gene amplifications or rearrangements, providing insights into the oncogenesis of SGN. Next-generation sequencing (NGS) is a high-throughput sequencing technology that allows for rapid and comprehensive analysis of genetic material and facilitates the identification of genetic alterations at a genome-wide level, aiding in the discovery of novel biomarkers and potential therapeutic targets for SGN [47,48]. These novel diagnostic tools have revolutionized the characterization of SGN by providing a clearer understanding of the cellular and molecular mechanisms underlying the disease. They have allowed for the identification of genetic alterations and biomarkers associated with SGN, shedding light on the oncogenesis of common SGN subtypes. Additionally, these tools have been used for predictive and prognostic purposes, helping to guide treatment decisions and improve patient outcomes (Table 2). Table 2 displays diagnostic, predictive, and prognostic markers in salivary gland neoplasms [49].

**Table 2:** Shows diagnostic, predictive, and prognostic markers in salivary gland neoplasm. Note: Adapted from Reference [49].

Tumor subtype	Genetic/molecular alterations	Role of alteration
Pleomorphic adenoma	PLAG1 alterations	Diagnostic
	HMGA2 alterations	Diagnostic
	HER2 overexpression	Predictive for therapeutic response
	AR overexpression	Predictive for therapeutic response
Acinic cell carcinoma	NR4A3 rearrangement	Diagnostic
Mucoepidermoid carcinoma	CRTC1–MAML2 fusion	Diagnostic/prognostic
	CRTC3–MAML2 fusion	Diagnostic/prognostic
Salivary duct carcinoma	AR gene alterations	Diagnostic/predictive for androgen-deprivation therapy response
	ERBB2 amplifications	Diagnostic/prognostic
	TP53, PIK3CA, H-RAS mutations	Diagnostic/prognostic (only TP53)
	Loss of heterozygosity of CDKN2A, p16, PTEN	Diagnostic
Secretory carcinoma	ETV6-NTRK3 fusion	Diagnostic
Clear cell carcinoma	EWSR1–ATR fusion	Diagnostic

## Conclusions

In conclusion, oncocytic tumors of the salivary glands are a rare and distinct group of neoplasms that exhibit unique morphological, immunohistochemical, and molecular features. Oncocytic cells, as well as the expression of particular markers such as MT, S100 protein, and vimentin, can help in the diagnosis of these tumors. Furthermore, the discovery of particular genetic abnormalities, such as mtDNA mutations, can provide valuable insights into the pathogenesis and potential therapeutic targets of oncocytic tumors. Further research is needed to better understand the biology and clinical behavior of these tumors, which will ultimately contribute to improved diagnosis and management strategies for patients with oncocytic tumors of the salivary glands.
